# Computational genomic identification and functional reconstitution of plant natural product biosynthetic pathways

**DOI:** 10.1039/c6np00035e

**Published:** 2016-06-20

**Authors:** Marnix H. Medema, Anne Osbourn

**Affiliations:** a Bioinformatics Group , Wageningen University , Wageningen , The Netherlands . Email: marnix.medema@wur.nl; b Department of Metabolic Biology , John Innes Centre , Norwich Research Park , Norwich , UK . Email: anne.osbourn@jic.ac.uk

## Abstract

Covering: 2003 to 2016

The last decade has seen the first major discoveries regarding the genomic basis of plant natural product biosynthetic pathways. Four key computationally driven strategies have been developed to identify such pathways, which make use of physical clustering, co-expression, evolutionary co-occurrence and epigenomic co-regulation of the genes involved in producing a plant natural product. Here, we discuss how these approaches can be used for the discovery of plant biosynthetic pathways encoded by both chromosomally clustered and non-clustered genes. Additionally, we will discuss opportunities to prioritize plant gene clusters for experimental characterization, and end with a forward-looking perspective on how synthetic biology technologies will allow effective functional reconstitution of candidate pathways using a variety of genetic systems.

## Abstract 2

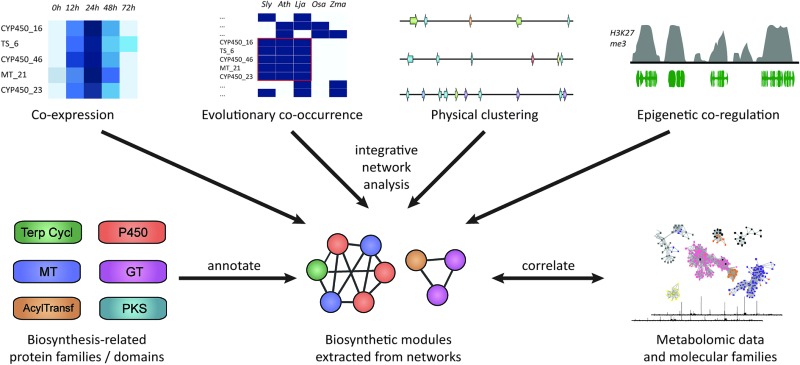
The last decade has seen the first major discoveries regarding the genomic basis of plant natural product biosynthetic pathways.

## 1. Introduction

Plants produce a huge array of natural products. Humans have relied on plants as sources of traditional medicines and drugs, dyes, colours, flavours, fragrances and agrochemicals throughout history. The compounds that have been exploited so far, however, represent only the tip of the iceberg in terms of the chemical diversity that is represented within the plant kingdom. Plant natural products are often lineage-specific, having evolved within narrow taxonomic groupings. They are normally produced only at certain growth stages in particular tissues, or in response to environmental stimuli (for example, challenge with pests, pathogens or elicitors), and are often found only at low concentrations within complex mixtures in plant extracts. These factors frequently cause problems in obtaining and/or cultivating the material and extracting and purifying the compounds of interest in useful quantities, for either research purposes or for commercial production. For these reasons, it difficult to access natural products from plants.

Identification of the genes involved in the biosynthetic pathways for the production of these molecules is a promising remedy, as it potentially allows heterologous expression of a pathway to acquire higher yields. Over seventy genome sequences have now been determined for different plant species and, in addition, a wealth of transcriptome data is available. Based on superficial analysis of these genomic resources, it is clear that plant genomes encode the capacity for an enormous amount of metabolic complexity. However, interpretation of this information and translation of predicted sequences into enzymes, pathways and products represents a major challenge. Systematic analysis of the metabolic capacity of particular plant species using transcriptomics and metabolomics has proved to be a very useful strategy for identifying candidate genes implicated in the biosynthesis of different types of natural products. If one or more genes encoding biosynthetic enzymes for the synthesis of a compound of interest have been identified, then this process can be guided by using the genes for characterized enzymes as bait in co-expression analysis, in concert with knowledge of the structure of the compound, to inform on the other enzymes that may be involved in the pathway.^1–3^ Alternatively, if candidate genes are identified solely on the basis that they are co-expressed and predicted to encode enzymes of specialized metabolism, but the pathway and the nature of the end product are unknown, then untargeted metabolomics can be a powerful tool to discover entirely new pathways and chemistries.^3^

In microbes, widely used bioinformatics-based approaches to discover new metabolic pathways are based on the identification of physically clustered groups of genes termed ‘biosynthetic gene clusters’ (BGCs).^4^ Intriguingly, it has recently been discovered that in plants the genes for a number of biosynthetic pathways are also encoded in operon-like gene clusters, which may facilitate co-regulation and stable co-inheritance.^5–7^ For the most part, these clustered pathways appear to have evolved relatively recently in evolutionary time within narrow taxonomic lineages and are not a consequence of horizontal gene transfer from microbes. This defies the assumption that gene ordering on plant chromosomes is more or less random. It also has an important practical implication: it potentially allows the straightforward identification of biosynthetic pathways from genome sequences, just like in bacteria and fungi. A recent computational study by Chae *et al.* ^8^ of the genomes of *Arabidopsis thaliana*, rice, soybean and sorghum indicated that genes associated with metabolism are indeed more often clustered than expected by chance in these species, and that the observed clusters of metabolic genes in *A. thaliana* and soybean are significantly enriched for specialized metabolism. In another recent study by Boutanaev *et al.*,^9^ it has been found that, in a larger number of plant genomes, the genes encoding terpene synthases and cytochrome P450s are frequently clustered. Nonetheless, it is still largely unclear how widespread the phenomenon of metabolic gene clustering is throughout the plant kingdom, how such clusters originate, and to what extent they are maintained during evolution.

**Fig. 1:**
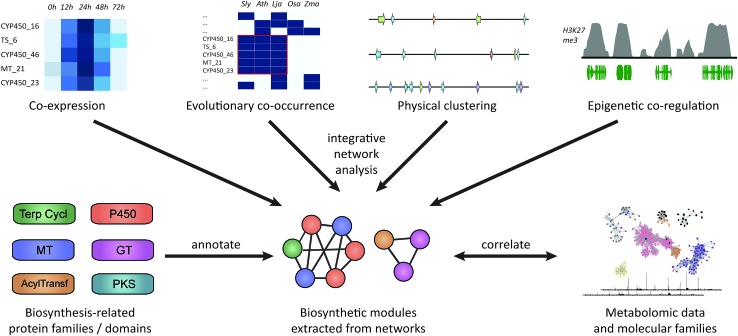
Approaches for plant biosynthetic pathway discovery. Physical co-clustering, co-expression, evolutionary co-occurrence, and epigenetic co-regulation can all be used to identify candidate biosynthetic pathways. Using, for example, network analysis, these approaches can also be combined, if sufficient data is available. Functionally cohesive modules can then be extracted from such a network and annotated for the presence of genes encoding biosynthesis-related protein families. Finally, modules that have a strong biosynthetic signature can be correlated to metabolite counts or molecular families derived from molecular networking^82,83^ of metabolite data.

Besides co-expression and genomic clustering, there are two other important strategies to identify biosynthetic pathways: evolutionary genomic approaches that use phylogenetic profiling to look at co-occurrence across genomes or that identify recent gene family expansions, and epigenomic approaches that harness shared patterns in chromatin-level regulation based on histone modification data (Fig. 1). In this review, we will discuss each of these four strategies, with respect to both chromosomally clustered and non-clustered pathways. Subsequently, we will discuss ways in which candidate pathways can be prioritized for experimental characterization, and how synthetic biology approaches can be applied to heterologously express the pathways to identify novel natural products.

## 2. Plant biosynthetic pathways: clustered and non-clustered

**Fig. 2:**
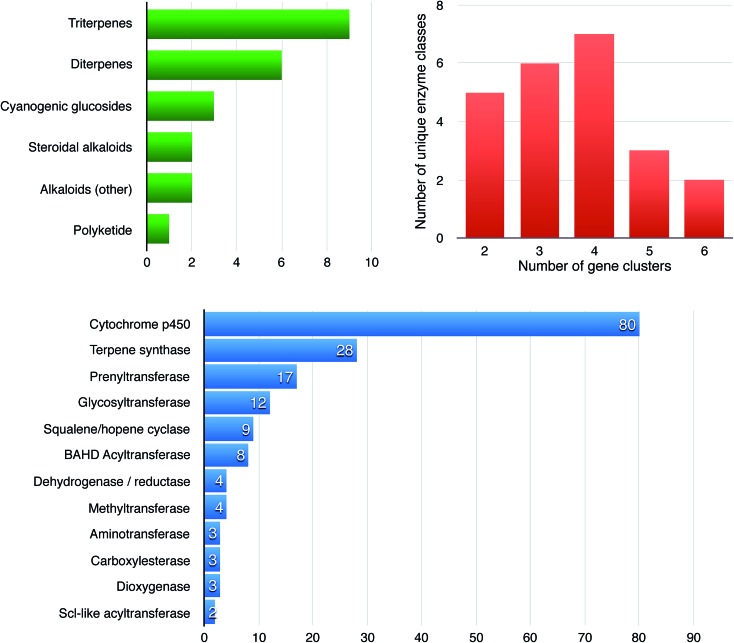
Features and statistics of 28 known plant biosynthetic gene clusters. The graphs show the distributions of compound classes produced from known enzymes encoded in plant biosynthetic gene clusters (green), the number of unique (broad) enzyme families per gene cluster (red) and the gene counts of enzyme families across all clusters (blue). The numbers for the latter two are based on automated annotation of broad enzyme families through the Pfam database;^84^ it should therefore be noted that any two enzymes from one Pfam protein family can still catalyze two significantly different chemical reactions. In all specific cases where only two enzyme classes are present in a cluster according to the figure, one of these comprises multiple distinct subclasses of cytochrome P450s belonging to at least two different P450 subfamilies.

More than two dozen plant biosynthetic pathways have now been shown to be encoded in gene clusters,^5–7^ which gives useful insights into the variations in cluster architecture (Fig. 2). The plant metabolic gene clusters reported so far range in size from ∼35 kb to several hundred kb and consist of three to ten genes. We define gene clusters as genomic loci that include genes for a minimum of at least three (and sometimes six or more) different types of biosynthetic reactions (*i.e.* genes encoding functionally different (sub)classes of enzymes).^5,7^ These distinctions are likely to turn out to be somewhat arbitrary as we learn more about the nature of metabolic gene clusters in plants and the birth, life and death of these forms of genomic organization.

A comprehensive picture of how widespread the phenomenon of gene clustering is in plants is still lacking. For several known molecules, the genes that encode their biosynthetic enzymes are not clustered. For example, the strigolactone biosynthesis genes *MAX1*, *MAX3*, *MAX4* and *AtD27* (ref. 10) are dispersed across three chromosomes of the *A. thaliana* genome, *MAX1* and *MAX3* being 8 Mb apart on chromosome 2, and *MAX4* and *AtD27* being located on chromosomes 4 and 1, respectively. Similarly, the biosynthetic pathways for glucosinolates,^11^ arabidopyrones,^12^ 4-hydroxyindole-3-carbonyl nitrile^3^ and camalexin^13^ in *A. thaliana* and the pathways for hydroxy-cinnamic acid amide,^14^ acylsugars^15,16^ and oxylipin^17^ in tomato show no or very limited clustering of their biosynthetic genes. Many of these pathways are non-linear, having branches to alternative end products, which, as we will see further below, might be one potential reason why no clustering is observed.

In some other cases, there is evidence of partial clustering of different types of pathway genes. For example, the genes encoding the enzymes for the first two steps in betalain biosynthesis (a CYP76 cytochrome P450 enzyme that coverts tyrosine to l-DOPA and an l-DOPA 4,5-dioxygenase) lie within 50 kb of each other in sugar beet (*Beta vulgaris*) on chromosome 2. A betalain regulatory locus (the R locus) is also linked to the CYP76 locus.^18^ The gene encoding the third step of the pathway, the UDP-glucosyltransferase cDOPA5GT, lies on chromosome 1 and is unlinked to these first two steps. This pathway does not therefore represent a clear-cut example of a clustered metabolic pathway. Nevertheless, the clustering of genes for two different steps in a metabolic pathway involving entirely different classes of enzymes is interesting. Terpene synthase and cytochrome P450 genes have also been found to be clustered in the conifer genome, suggesting that these types of association may have some functional significance.^19,20^ Another good example of partial clustering is the monoterpene indole alkaloid biosynthetic pathway from *Catharanthus roseus*.^21^ This pathway has been shown to involve multiple cases where two different steps are encoded on the same genomic locus; in fact, this local clustering greatly aided the elucidation of some steps in the pathway. It is not fully certain yet, however, whether or not some of these loci are part of one or more larger gene clusters, as the current genome assembly is still quite fragmented.

**Fig. 3:**
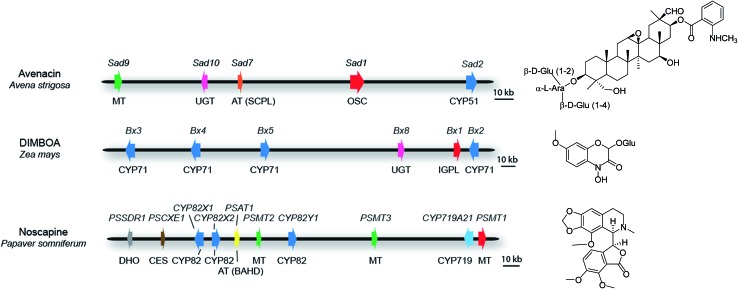
Some examples of plant metabolic gene clusters. Genes are indicated by arrows and gene(s) for the first committed pathway step are indicated in red. Gene names are indicated above the clusters and class of biosynthetic enzyme below. Abbreviations: OSC, oxidosqualene cyclase; IGPL, indole 3-glycerol phosphate lyase; AT (SCPL), SCPL-acyltransferase; AT (BAHD), BAHD-acyltransferase; MT, methyltransferase; UGT, UDP-dependent sugar transferase; DHO, dehydrogenase/reductase; CES, carboxylesterase; CYP, cytochrome P450. The oat avenacin cluster contains five genes for the synthesis, oxidation and acylation of the triterpene scaffold.^31–34^ Two other loci (*Sad3* and *Sad4*) have been shown to be required for avenacin glucosylation but not yet cloned. *Sad3* lies within 3.6 cM of the core cluster while *Sad4* is unlinked.^35,85^ The maize DIMBOA pathway includes three genes that are not shown in the figure; *Bx7*, which is separated from the core cluster by an intervening region of 15 Mb; the sugar transferase gene *Bx9*, which is located on a different chromosome; finally a further gene *Bx6* is not shown because its genomic location has not yet been established.^27–30^ The noscapine cluster from poppy contains all of the pathway genes except the gene for tetrahydroprotoberberine *cis-N*-methyltransferase (TNMT), which catalyses the first committed pathway step.^26^

For those clusters that meet the definition of a plant natural product biosynthetic gene cluster (see above), some contain all of the pathway genes. Examples include the thalianol and marneral clusters in *A. thaliana*, which consist of four and three genes, respectively,^22,23^ as well as the three-gene cyanogenic glycoside clusters in sorghum, *Lotus japonicus* and cassava, each of which has evolved independently.^24^ In barley, a three-gene cluster has been identified that is necessary and sufficient for the biosynthesis of polyketide diketones that determine the *Cer-cqu* waxy leaf phenotype.^25^ In other cases, while the core cluster contains most of the genes in the pathway, there are some anomalies. For example, the ten-gene cluster for the synthesis of the medicinal benzylisoquinoline alkaloid noscapine in poppy (*Papaver somniferum*; Fig. 3) contains all of the pathway genes except the gene for a *cis-N*-methyltransferase (TNMT).^26^ Since the genome sequence of poppy is not available, this cluster was defined by assembly of a bacterial artificial chromosome (BAC) contig spanning the genes encoding the pathway enzymes. Although the location of the *TNMT* gene has not been absolutely defined, *TNMT* gene homologues are present in the flanking regions of the sequenced cluster region. In other cases some of the pathway genes are less tightly linked to the main cluster region and there may be intervening genes with no obvious function in specialized metabolism between these ‘peripheral’ genes and the core cluster. For example, seven of the genes for the biosynthesis of benzoxazinoids in maize form a cluster on chromosome four^27–30^ (Fig. 3). A further pathway gene encoding an *O*-methyltransferase (*Bx7*) is loosely linked, lying within 15 Mb of the core cluster. The core cluster contains a gene encoding the sugar transferase Bx8, which is required for benzoxazinoid glucosylation. A *Bx8* homologue *Bx9*, encoding an enzyme that also has activity towards benzoxazinoids, is located on a different chromosome, although it is not known whether Bx9 is a *bona fide* part of the benzoxazinoid pathway in maize. In diploid oat (*Avena strigosa*), five genes for the biosynthesis of the antimicrobial triterpene glycoside avenacin are located within a 200-kb region that does not contain any other obvious intervening genes (Fig. 3). These genes encode enzymes required for the synthesis, oxidation and acylation of the triterpene scaffold.^31–34^ Two other loci (*Sad3* and *Sad4*) have been shown by mutation to be required for avenacin glycosylation but have not yet been cloned.^35^ *Sad3* is loosely linked to the avenacin cluster (within 3.6 centimorgans); *Sad4* is unlinked. However, *Sad4* is required for glycosylation of other compounds in addition to avenacins and is not absolutely required for avenacin biosynthesis. Sad4 therefore appears to be ‘moonlighting’ and is not an integral part of the pathway. The above examples illustrate the importance of having rigorous support for the involvement of candidate pathway components in the synthesis of particular natural products in plants. *In vitro* assays of enzyme function can be misleading. Enzymatic information generated *in vitro* may therefore result in inappropriate implication of enzymes that are not dedicated pathway components.

Examples of metabolic gene clusters that contain genes encoding at least three different types of metabolic enzymes are given above. In some cases, the cognate pathways also involve one or more steps encoded by occasional ‘peripheral’ pathway genes, although as stated above the interpretation of which genes are *bone fide* pathway genes and which ones encode moonlighting enzymes that are not genuine pathway components requires some consideration. In other cases, there is a core cluster of genes encoding pathway enzymes but there are also additional ‘mini-clusters’ of up to two genes encoding steps required for elaboration of the pathway scaffold.^7^ Examples of the latter include the pathways for the biosynthesis of steroidal glycoalkaloids in tomato and potato. In tomato, most of the genes for steroidal glycoalkaloid biosynthesis are clustered on chromosome seven. Two further pathway genes are adjacent to each other on a different chromosome. A similar situation exists in the close relative of tomato, potato, where regions syntenic to those in tomato contain the genes required for the biosynthesis of the steriodal alkaloids alpha-chaconine and alpha-solanine.^36^ Similarly, in cucumber (*Cucumis sativus*) five genes encoding the enzymes required for the biosynthesis of triterpene glycosides associated with bitterness (cucurbitacins) are clustered on chromosome six, while four other genes are located elsewhere in the genome – a CYP71 and two CYP88 genes are co-located on chromosome three, and a CYP87 gene on chromosome one.^37^

Finally, things are also complicated by the fact that many complex plant genome assemblies are either not available or highly fragmented. For highly fragmented genome sequences, clustering will not be evident if the genes are separated on different contigs in the assembly. Where genome sequences are not available and analysis has relied on exploitation of transcriptome data, it is simply not known whether pathway genes are clustered or not. For example, the steps in the mayapple podophyllotoxin pathway^2^ have been elucidated but the genome sequence has not been determined. Similarly, the artemisinin pathway has been well characterized but the genome sequence of the producing plant *Artemisia annua* is not available. As more (complex) plant genomes are sequenced, we will learn more about the diversity and variation of biosynthetic gene clustering in plants.

## 3. Computational identification of plant biosynthetic pathways

### 3.1 Identifying plant gene clusters

For the discovery of clustered pathways, much can be learned from previous work on bacterial and fungal biosynthetic pathway discovery. Various algorithms for the identification of biosynthetic gene clusters have been designed for use on bacterial and fungal genome sequences.^38–45^ However, the implemented detection logic for these algorithms makes several assumptions that do not hold true for plant genomes. Specifically, most assume that (1) every time a gene encoding a ‘scaffold-generating’ enzyme such as a polyketide or terpene synthase is identified in a genome, it will be surrounded by a gene cluster and that (2) all clustered groups of biosynthetic genes in a genome will encode a multi-step biosynthetic pathway. On the contrary, some plant genes that encode scaffold-generating enzymes occur either as singletons (without a surrounding gene cluster) or in tandem arrays of nearly identical copies that do not encode subsequent enzymatic steps in a pathway. To further complicate things, some tandem arrays, such as the array of Bx2-3-4-5 cytochrome P450-encoding genes in the DIMBOA gene cluster^27^ in fact do encode subsequent steps in a pathway that have evolved through multiple iterations of duplication and divergence.

Also, many (but not all^46^) microbe-oriented algorithms assume that all biosynthetic gene clusters will contain a gene encoding a ‘scaffold-generating’ enzyme (which, for example, produces a peptide, polyketide or terpene backbone). However, some plant ‘scaffold-generating’ reactions are in fact modifications of primary metabolic ‘scaffolds’ such as amino acids^47^ and are therefore not necessarily linked to one specific ‘scaffold-generating’ enzyme family. A further complication in the process of identifying plant biosynthetic gene clusters is that in some cases the pathways are split over multiple loci, such as in the cases of tomatine/solanine^36^ and cucurbitacin;^37^ hence, even when a given biosynthetic pathway involves a scaffold-generating enzyme such as a terpene synthase, not all loci that code for steps of that pathway will necessarily encode one.

To overcome these challenges, algorithms for the detection of plant biosynthetic pathways should aim to identify *all* genes encoding biosynthetic enzymes (*i.e.*, as many as possible), instead of just those encoding the scaffold-generating enzymes. For the identification of plant gene clusters, this would require constructing a carefully curated and comprehensive catalogue of sequence models (*e.g.*, profile hidden Markov models^48^) for the detection of enzyme-coding genes involved in specialized metabolism. Additionally, when encountering genomic loci encoding enzymes of the same enzyme superfamily, intelligent checks should be implemented to evaluate whether these enzymes are sufficiently different from one another to be likely to catalyze different reactions, for example by evaluating their overall amino acid sequence similarity or even the similarity of amino acids surrounding the enzyme active site (based on known structural models for homologous proteins): while it is possible that two terpene synthases with 90% amino acid sequence identity and identical active site residues have functionally diverged through changes in only one or two crucial amino acids, the presence of different catalytic functions would be much more likely if they only had 30% sequence identity and major differences in their active sites. Also, detailed computational subclassification of broad enzyme superfamilies such as cytochrome P450s into their constituent families would greatly help to both identify the presence of distinct subclasses in a genomic locus and to predict their potential functions in the encoded biosynthetic pathway.^7^

Besides biological differences between plants and microbes, there are also practical differences: for example, plant genes can have very complex intron–exon structures that make it difficult to correctly predict the protein-coding regions of a genome. Most of the time, this problem is overcome by making use of RNA-Seq datasets to identify the exons; however, many biosynthetic pathways (*e.g.*, for defense-related compounds) are not expressed under typical conditions, which potentially leads to large-scale misannotations of precisely such important genomic features. Especially for non-model plants, it might therefore be important to, *e.g.*, evaluate multiple possible gene models when identifying the presence of key biosynthetic domains in a genomic region.

Regardless of the important differences between microbial and plant biosynthetic gene clusters, many of the principles found in tools like antiSMASH^40–42^ can potentially be adapted to the unique properties of natural product biosynthesis in the plant kingdom. These principles include, *e.g.*, (1) the detection of enzyme families through the use of profile hidden Markov models,^48^ constructed from multiple sequence alignments, that capture the sequence diversity of a protein family and can be used to recognize additional members of such a family, (2) the use of pattern matching and machine learning to assess enzyme active sites based on 3D information from crystal structures,^49^ which can be used to predict activity and substrate specificity of key biosynthetic enzymes (although a meta-analysis of plant terpene synthases in 2009 found few correlations between residues and catalytic mechanisms^50^), and (3) comparative genomic analysis of gene clusters in different organisms^51,52^ to infer functions of conserved parts of biosynthetic pathways from homology.

### 3.2 Using epigenetics and co-expression analysis to identify clustered and unclustered pathways

The genes for specialized metabolic pathways in plants are under strict regulatory control. Specialized metabolites are often synthesized only in particular cell types, at certain developmental stages and/or in response to environmental triggers such as pest and pathogen attack or elicitor treatment. Consistent with this, Omranian *et al.* ^53^ found that, in the data of Chae *et al.*,^8^ genes for specialized metabolic pathways are more often coexpressed than genes encoding enzymes involved in non-specialized metabolism (having an assortativity statistic—which measures to which extent the corresponding nodes in a coexpression network are connected—of 0.118 *versus* 0.066, *P* < 0.001). Clearly, the co-ordinate transcription of genes depends on their availability to pathway-specific transcription factors. The organization of pathway genes in physically linked metabolic clusters has the potential to provide an additional higher level of regulation above and beyond that of unlinked pathways through condensation/decondensation of localized cluster-wide chromatin domains.^5–7^ When the chromatin encompassing a cluster is decondensed into euchromatin, the pathway genes will become accessible for transcription. In contrast, when the cluster region is sequestered in condensed heterochromatin, it will be inaccessible, thus providing protection against ectopic expression and production of potentially toxic metabolites and pathway end products. Such a mechanism could be regarded as a ‘belt and braces’ approach to the regulation of these recently evolved and highly insulated clustered pathways – a way of dealing with dangerous new chemistry.

Several lines of evidence indicate that plant metabolic gene clusters are subject to regulation at the level of chromatin. DNA fluorescence *in situ* hybridization (DNA FISH) using probes for genes within the oat avenacin cluster indicates that the cluster region undergoes chromatin decondensation when in its active form.^54^ Investigation of the regulation of the thalianol and marneral clusters in *A. thaliana* using chromatin mutants and chromatin immunoprecipitation (ChIP) has implicated two major chromatin markings in cluster regulation: the histone 2 variant H2A.Z in cluster activation, mediated by the SWR1 chromatin remodeling complex;^55^ and polycomb-mediated histone H3 lysine 27 trimethylation (H3K27me) in cluster repression.^7^ Thus, H2A.Z and H3K27me3 appear to be involved in a dynamic transition between different chromatin environments associated with the active and inactive states of the clusters. Biosynthetic gene clusters from oat, maize and rice also have pronounced H3K27me3 marking when in their ‘off’ state, suggesting that polycomb-mediated cluster repression occurs in both eudicots and monocots. These chromatin markings delineate the clusters and are discrete, encompassing the biosynthetic genes but not the immediate functionally unrelated flanking genes. Knowledge of these hallmark features has opened up new strategies for genome mining and has enabled the discovery of novel biosynthetic gene clusters.^56^ In contrast, the genes of non-clustered plant biosynthetic pathways examined so far do not have pronounced H3K27me3 or H2A.Z markings.

**Fig. 4:**
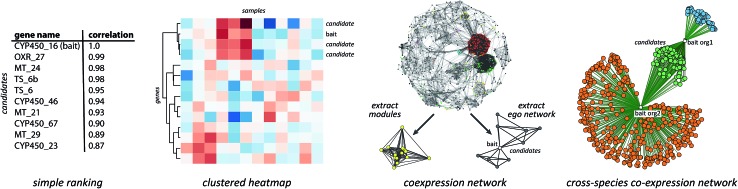
Co-expression techniques to identify biosynthetic pathway components. The simplest way to identify novel candidates for a pathway is to use a bait gene that is known to be involved in the pathway and to rank all other genes by correlation coefficient to the bait. In order to also visualize the interrelationships between all (relevant) genes, clustered heatmaps can be used. The same is true for coexpression networks, which have the added advantage that they can also be used in ‘untargeted’ approaches to identify candidate pathways by extracting modules out of the network without using a bait. Finally, cross-species co-expression networks can be used to identify orthologous groups of genes whose co-expression is conserved over longer evolutionary periods.

It is not yet clear whether the genes within individual clustered specialized metabolic pathways are more highly co-expressed than those of non-clustered specialized metabolic pathways. In any case, co-expression studies (Fig. 4) have contributed greatly to the elucidation of non-clustered pathways. For example, the 4-hydroxyindole-3-carbonyl nitrile pathway in *A. thaliana* was recently elucidated by using a single cytochrome P450-encoding gene as ‘bait’ to identify other genes that were strongly co-expressed with it.^3^ In a similar way, the podophyllotoxin pathway from mayapple was identified in the same lab,^2^ although in this case genome sequence information is not available in order to know whether the pathway is clustered or not.

Besides co-expression analysis using bait genes or co-expression clustering, co-expression networks are also a popular tool to identify expression patterns that can lead to the discovery of biosynthetic pathways.^87^ In such a network, each gene is represented by a node, and the nodes of genes whose expression shows a correlation above a certain threshold are connected by an edge. If metabolomic data is available from the same experiment, metabolites can also be added to the same network with a separate node type. The cutoff that is used to determine which connections should be shown in such a network is often arbitrary, but an approach like random matrix theory^57^ can be used to rationalize this. The disadvantage of co-expression networks is that, in many experiments, large numbers of genes are correlated to each other, leading to a big ‘hairball’ that is difficult to interpret. This can be alleviated by, for example, first performing differential expression analysis on a biological induction or tissue where certain pathways are expected to be overexpressed, and then generating a co-expression network for only the differentially expressed genes. Alternatively, the complexity of the network can be reduced by only considering genes with protein domains related to specialized metabolism. Another option is to use the bait gene approach within the network to extract ‘ego networks’: a first-order ego network, for example, contains all the direct neighbours in the network of the bait gene, while a second-order ego network will also contain the direct neighbours of these neighbours. Finally, algorithms like weighted correlation network analysis (WGCNA),^86^ the Markov Cluster algorithm (MCL)^58^ or Infomap^59^ can be used to break up a complex network into small clusters that can be individually studied for the presence of biosynthesis-related domains or correlated with metabolite absence/presence under the same conditions.

Normally, a co-expression network is constructed based on expression data from a range of different tissues and/or biological or physical treatments. An alternative to this, however, is to exploit the evolutionary variation in expression between species: during evolution, genes involved in the same pathway are expected to remain co-expressed, while the expression of other genes and pathways are expected to slowly diverge from it. When detailed orthology predictions are made for all the genes in multiple related genomes, they can be combined in a cross-species co-expression network to aid in pathway identification. The gene clusters for α-tomatine and α-solanine in tomato and potato were discovered based on such a cross-species co-expression analysis, where it was found that a range of genes co-expressed with a bait gene were clustered on two chromosomes of each of the species.^36^ In plant taxa with many sequenced genomes and transcriptomes, such as the Solanaceae and Brassicaceae, such an analysis could also be potentially applied across many species at once. An online tool, CoExpNetViz,^60^ has recently been published that can be used for bait-driven analysis of such cross-species networks.

### 3.3 Evidence from evolutionary genomics for pathway prediction

Another interesting and largely unexploited opportunity to predict functional connections between biosynthetic genes is the use of evolutionary genomic analysis. After all, genes whose products are involved in the same biochemical process are expected to co-evolve in terms of gain/loss events. For years, phylogenetic profiling techniques^61^ have exploited this feature to predict biological pathways from absence/presence matrices of genes/proteins across large numbers of species. Recently, an advanced version of such an algorithm was published (termed ‘CLIME’), which uses a tree-structured hidden Markov model to infer the evolutionary histories of genes and to subsequently predict evolutionarily conserved multi-gene modules based on this.^62^ Given the way the algorithm works, it will probably be most effective to predict (or aid in the prediction of) linear multi-enzyme pathways that are either entirely present or entirely absent in most species. If a pathway is more branched and has many biochemical variations across species, theory predicts that this will likely blot out the absence/presence-based signal. However, even for more branched pathways, evolutionary analysis can be very useful: if it is known that certain metabolites are taxon-specific, it is expected that at least some of the corresponding biosynthetic enzymes will also be encoded by taxon-specific genes. This is exemplified by how the caffeine biosynthetic pathway was largely uncovered thanks to the discovery of a species-specific gene family expansion of *N*-methyltransferases,^63^ which through duplication and divergence allowed precursors to be transformed into this well-known tri-methylated compound. Similarly, ‘blooms’ of cytochrome P450 diversification have been associated with the evolution of specialized metabolic diversity.^64^ For the study of species for which genome and metabolite information of close relatives is available, this principle could potentially be utilized in an automated manner for multiple natural products in parallel. While the information by itself might not be sufficient to unequivocally pinpoint a gene-metabolite connection, it does have significant potential to add statistical power to clustering-based or co-expression-based approaches when included in an integrative bioinformatic framework (Fig. 1).

## 4. Prioritization and functional reconstitution of plant pathways

### 4.1 The need to prioritize

Several advances in sequencing technologies are currently making it increasingly affordable and straightforward to sequence complete plant genomes. Long read technologies like those of Pacific Biosciences and artificial long read technologies like Illumina TruSeq and 10X Genomics offer large improvements in the ability to close complex plant genomes.^65^ Moreover, recently revived optical mapping technologies such as those from BioNano Genomics potentially allow scaffolding of resulting contigs to close chromosomes from end to end. Accordingly, a high-quality *de novo* assembly of a medium-sized plant genome can now be obtained for merely $30–50k, which is a fraction of past prices. As further technological improvements are still rapidly ongoing, it should soon be feasible to sequence genomes from hundreds and thousands of plant species, including a wide range of plants that have been used as herbal medicines for centuries. These will encode very large numbers of biosynthetic pathways and gene clusters, which means that computational tools will be of great importance to classify and cluster these pathways and to prioritize them for experimental characterization to drive natural product discovery. One powerful technique that has been successfully used to navigate through large numbers of biosynthetic pathways in bacteria is the use of similarity networks and pathway family reconstruction.^46,66,67^ These techniques make it possible to effectively visualize biosynthetic diversity, identify novel classes of pathways and correlate pathways to metabolomic and phenotypic data. Similar tools could be developed for predicted plant pathways, by adapting the single-locus-based approaches from bacteria to accommodate both single-locus and multi-locus pathways predicted to be encoded in plant genomes. At the same time, the expected accumulation of sequenced genomes will allow unprecedented opportunities to analyze the evolution of biosynthetic pathways and gene clusters, in order to understand how the stunning molecular diversity of plant chemistry worldwide has come about over millions of years.

### 4.2 Synthetic biology technologies for functional reconstitution of candidate pathways

**Fig. 5:**
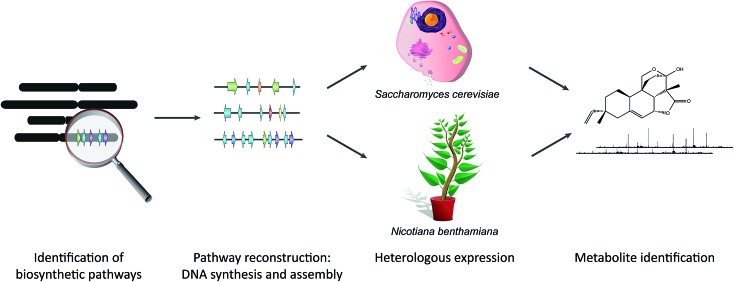
Synthetic biology approaches to characterize plant biosynthetic pathways. For identified (candidate) pathways, a construct is synthesized and assembled that contains all genes needed to produce the end product of the pathway, as well as the required regulatory elements. The construct is then expressed in either yeast or tobacco, after which the metabolite is identified and further characterized.

Rapid advances in sequencing technology, the availability of an ever-growing number of genome sequences from diverse plant species, and the application of powerful computational tools for discovery of biosynthetic gene clusters is expected to yield huge numbers of candidate pathways that will require functional validation. Synthetic biology technologies (Fig. 5) are well equipped to deal with this. The genes of new predicted biosynthetic clusters can be synthesized by commercial DNA synthesis companies and the cost of DNA synthesis is likely to decrease going forwards. DNA assembly methods that enable multiple parts to be assembled in a single reaction mean that construction of expression vectors is no longer a rate-limiting step. Assembly in yeast normally relies on overlap-dependent recombination. Golden Gate assembly methods based on Type IIS restriction enzymes have been widely adopted for plant synthetic biology and a common syntax has been proposed to enable exchange of compatible DNA parts.^68–73^ Highly complex pathways for the synthesis of plant natural products have been reconstructed successfully in yeast, including those for the synthesis of artemisinic acid from wormwood, opioids from poppy and the monoterpene indole alkaloid strictosidine from Madagascar periwinkle.^74–76^ Plant-based expression systems have the potential to overcome problems that may be encountered with post-translational processing, subcellular localization, precursor supply, sequestration and toxicity in yeast. Transient expression in leaves of the wild tobacco species *Nicotiana benthamiana* is proving to be a highly effective system for expression of single and multiple biosynthetic pathway genes.^77–81^ This method involves infiltrating *N. benthamiana* leaves with *A. tumefaciens* containing the appropriate expression vectors and is very rapid, yielding results within around a week.

## 5. Conclusions

The discover-build-test cycle for plant biosynthetic pathways and gene clusters will inevitably accelerate with further technological advances and access to a rapidly growing number of plant genome sequences, including for medicinal plants. Computational genomic analysis will be particularly important in enabling the vast metabolic potential of plant genomes to be unlocked by opening up doors that lead to previously unexplored reservoirs of new enzymes, pathways and chemistries. This rapidly growing body of knowledge will feed back into and inform the continued development of computational tools for genomics-based natural product discovery in plants.
